# Supplementary material for: Concise and Free-Metal Access to Lactone-Annelated Pyrrolo[2,1-a]isoquinoline Derivatives via a 1,2-Rearrangement Step
Source: Int J Mol Sci. 2024 Jan 16;25(2):1085. doi: 10.3390/ijms25021085 (PMC10816086; doi:10.3390/ijms25021085)
Supplement: Supplementary file 1 [file ijms-25-01085-s001.zip › ijms-2822305-supplementary.pdf]

Supporting Information

Concise and Free-Metal Access to Lactone Annelated Pyrrolo[2,1-*a*]isoquinoline Derivatives via a 1,2-Rearrangement Step

Arina Y. Obydennik [1], Alexander A. Titov [1], Anna V. Listratova [1], Tatiana N. Borisova [1], Victor B. Rybakov [2], Leonid G. Voskressensky [1,*] and Alexey V. Varlamov [1]

[1] *Organic Chemistry Department, Science Faculty, Peoples' Friendship University of Russia (RUDN University), 6 Miklukho-Maklaya Street, Moscow 117198, Russia*

[2] *Department of Chemistry, Lomonosov Moscow State University, Leninskie Gory, 1-3, Moscow 119991, Russia*

✉ Leonid G. Voskressensky
voskresenskiy-lg@rudn.ru

Table of content

X-ray Structure Determination of Compound 3a 2

Copies of ^{1}H and ^{13}C NMR spectra 3

Figure S1. NOESY spectrum of **6b** in $CDCl_3$. 16

Figure S2. HMQC ^{1}H-^{13}C spectrum of **6b** in $CDCl_3$. 16

Figure S3. HMBC ^{1}H-^{13}C spectrum of **6b** in $CDCl_3$. 16

Table S1

X-ray Structure Determination of Compound 3a

Single crystal of **3a** was grown from MeOH at room temperature.

**Compound**	**3a**
Empirical formula	$C_{19}H_{21}NO_6$
Formula weight	359
Temperature/K	295
Crystal system	triclinic
Space group	P $\bar{1}$ (2)
a, Å	7.6475(7)
b, Å	9.1412(9)
c, Å	13.9957(11)
α, °	104.356(4)
β, °	92.863(4)
γ, °	108.263(4)
Volume, $Å^3$	891.399
Z	2
R-Factor (%)	4.81
deposit CCDC	2156399

Copies of ^{1}H and ^{13}C NMR spectra

Methyl (2*E*)-3-[6,7-dimethoxy-1-(3-methoxy-3-oxoprop-1-yn-1-yl)-1-(4-nitrophenyl)-3,4-dihydroisoquinolin-2(1*H*)-yl]prop-2-enoate 1g

^{1}H NMR (600 MHz, $CDCl_3$):

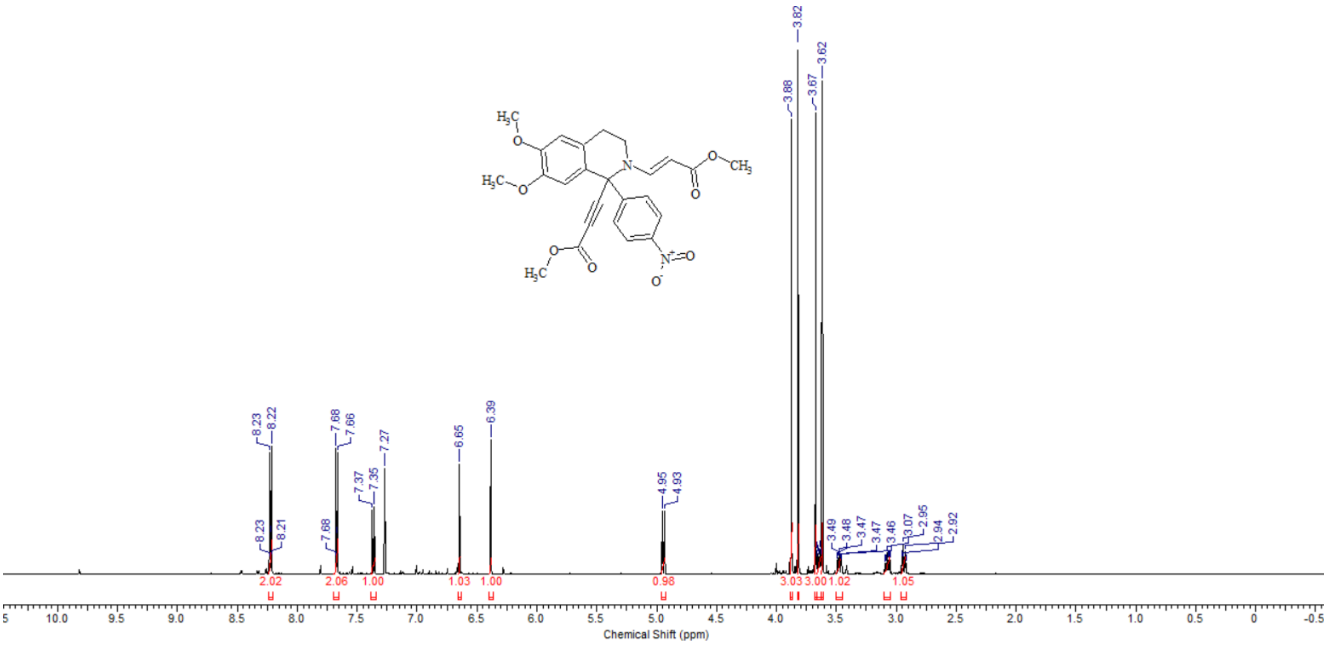

^{13}C NMR (150 MHz, $CDCl_3$):

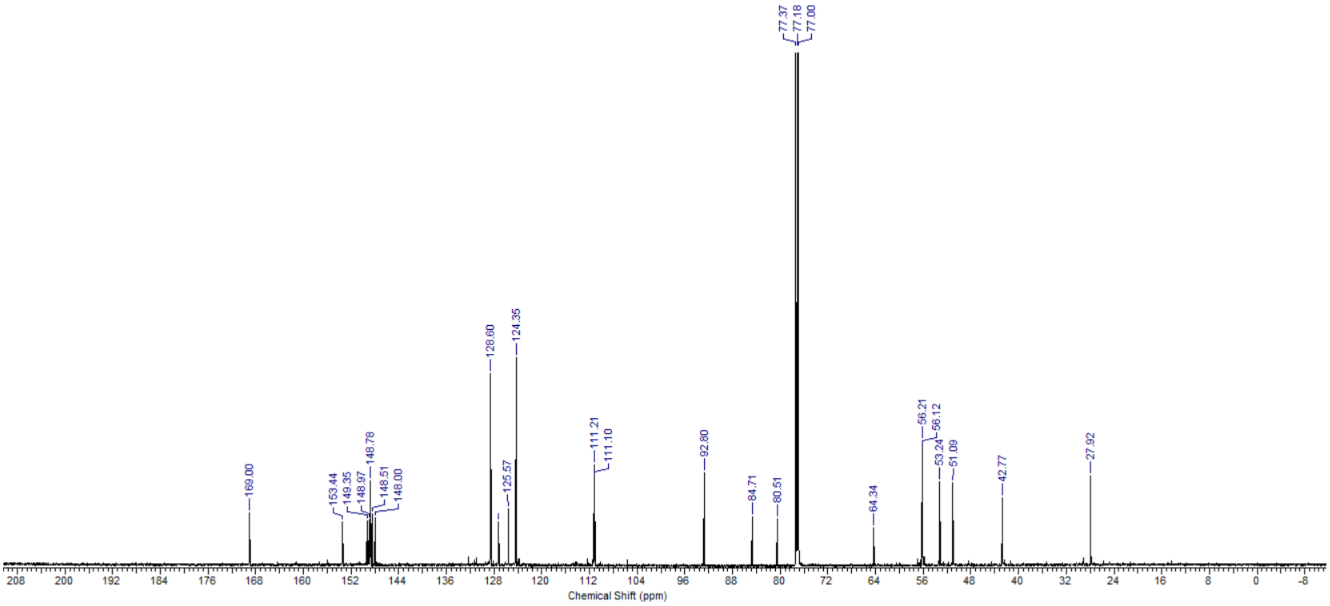

Dimethyl 11b-(4-nitrophenyl)-9,10-dimethoxy-7,11b-dihydro-6*H*-pyrido[2,1-*a*]isoquinoline-2,3-dicarboxylate 2g

^{1}H NMR (600 MHz, $CDCl_3$):

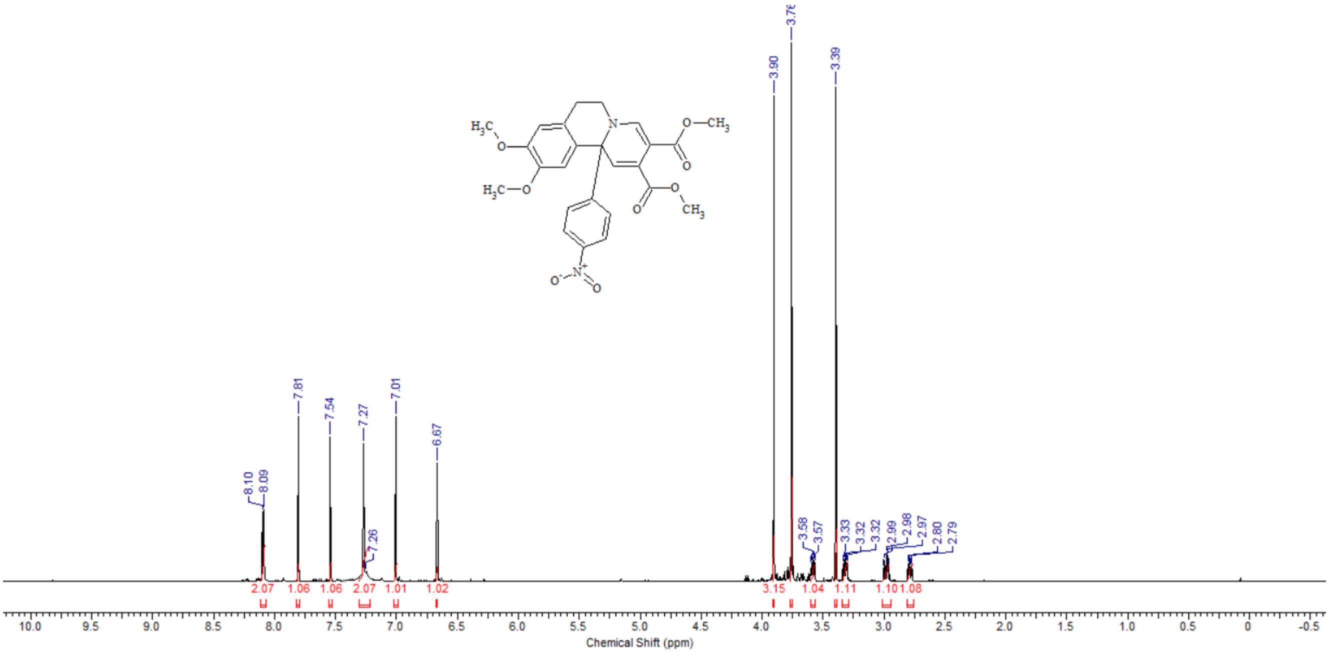

^{13}C NMR (150 MHz, $CDCl_3$):

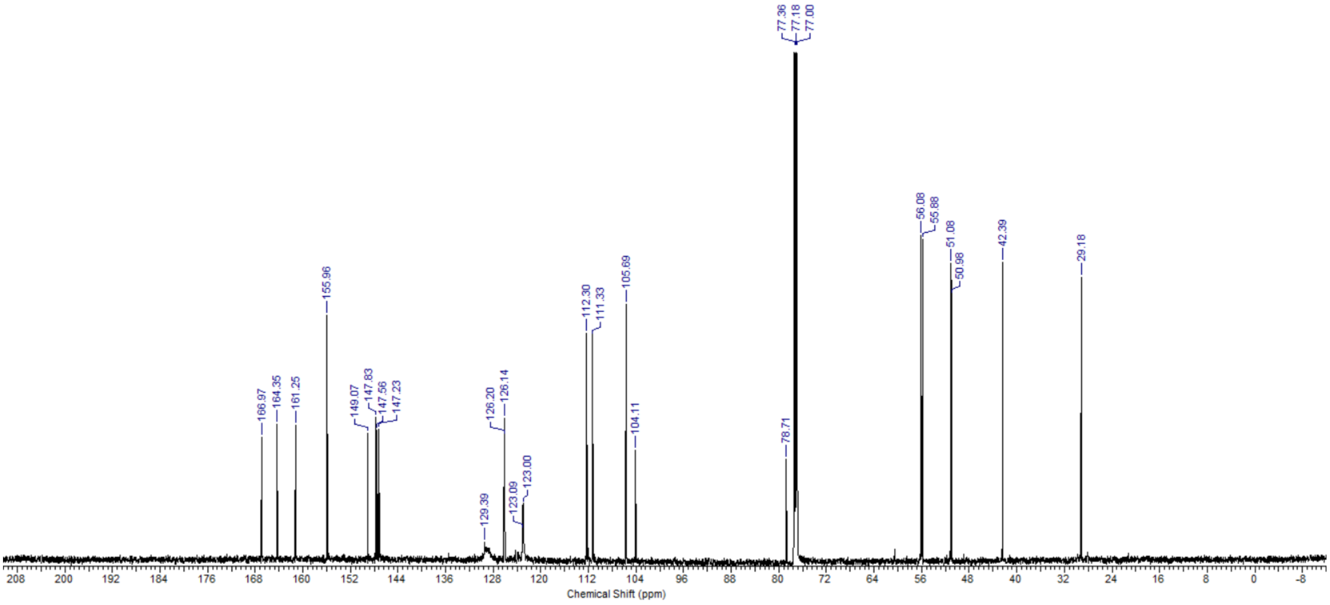

Methyl 10,11-dimethoxy-3a-methyl-2-oxo-3,3a,7,8-tetrahydro-2*H*-furo[2’,3’:2,3]pyrrolo[2,1-*a*]isoquinoline-4-carboxylate 3a

^{1}H NMR (600 MHz, $CDCl_3$):

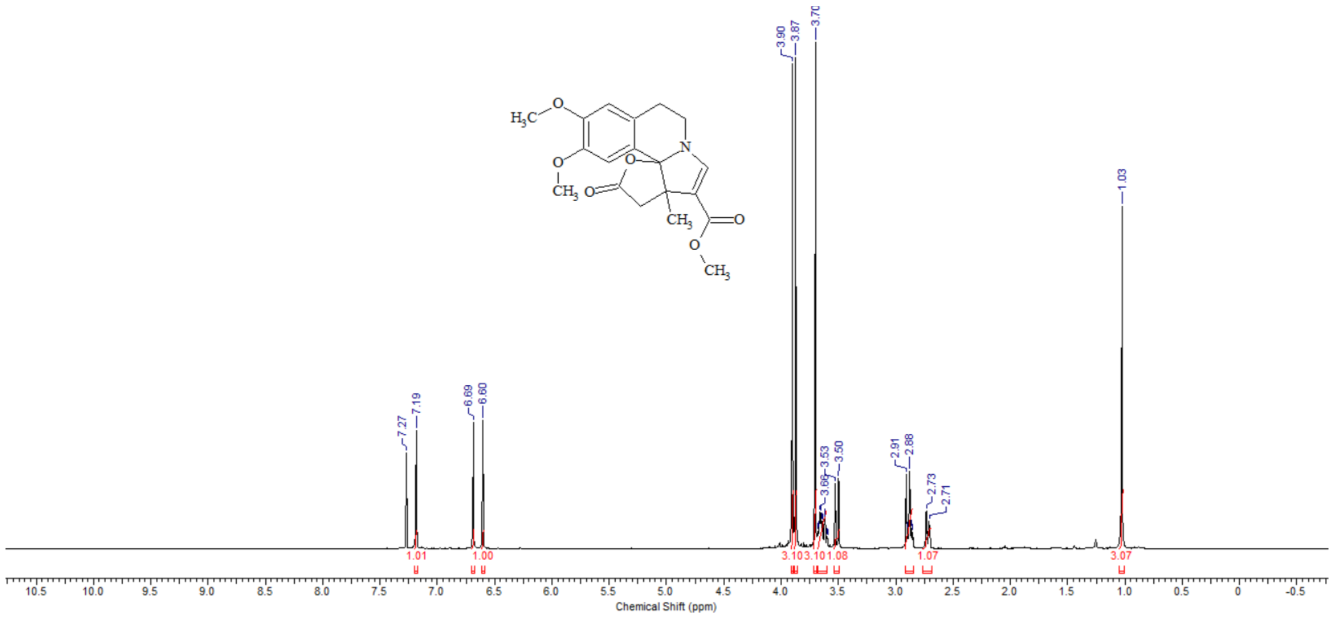

^{13}C NMR (150 MHz, $CDCl_3$):

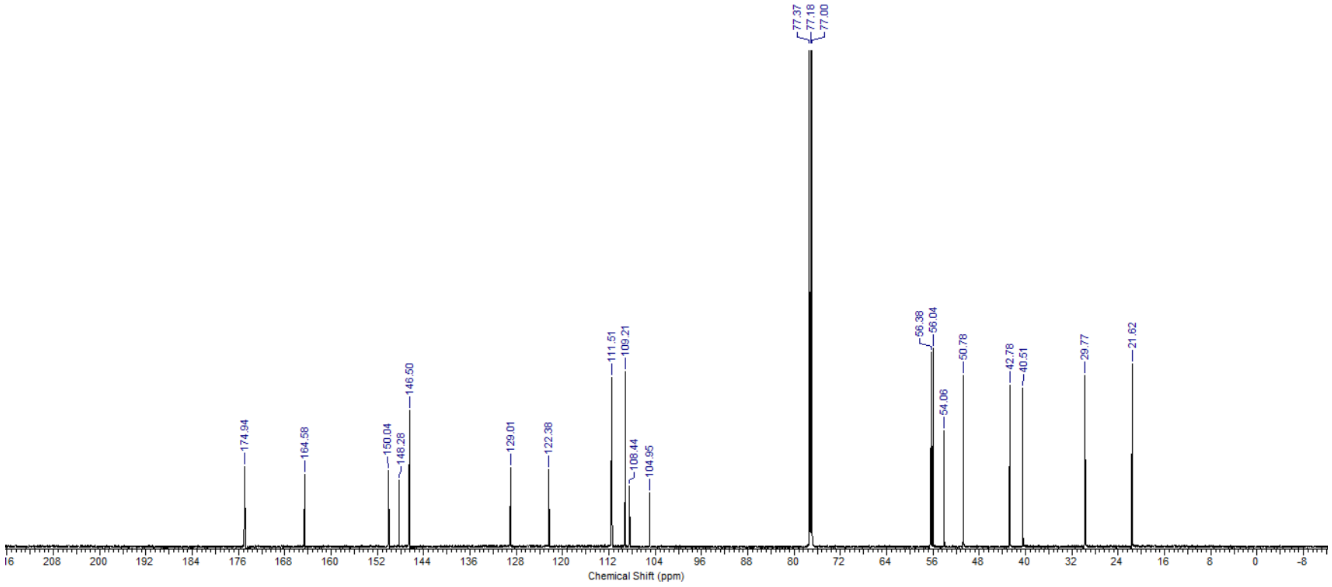

Methyl 10,11-dimethoxy-2-oxo-3a-(propan-2-yl)-3,3a,7,8-tetrahydro-2*H*-furo[2',3':2,3]pyrrolo[2,1-*a*]isoquinoline-4-carboxylate 3b

^{1}H NMR (600 MHz, $CDCl_3$):

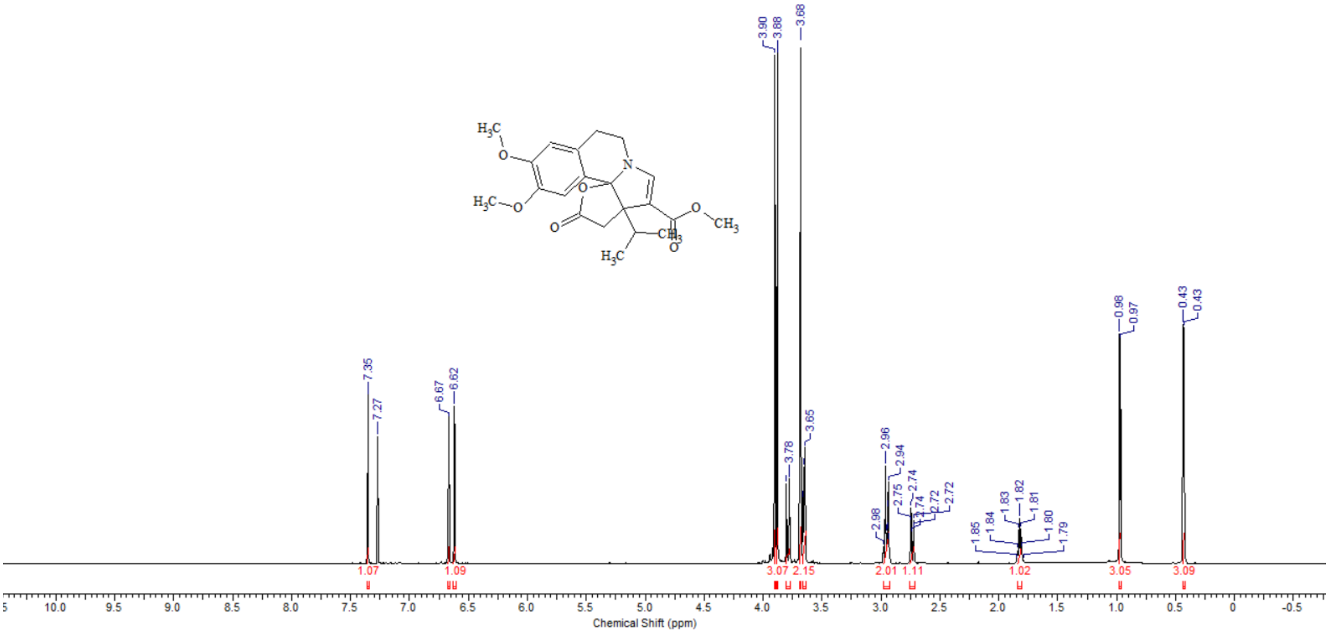

^{13}C NMR (150 MHz, $CDCl_3$):

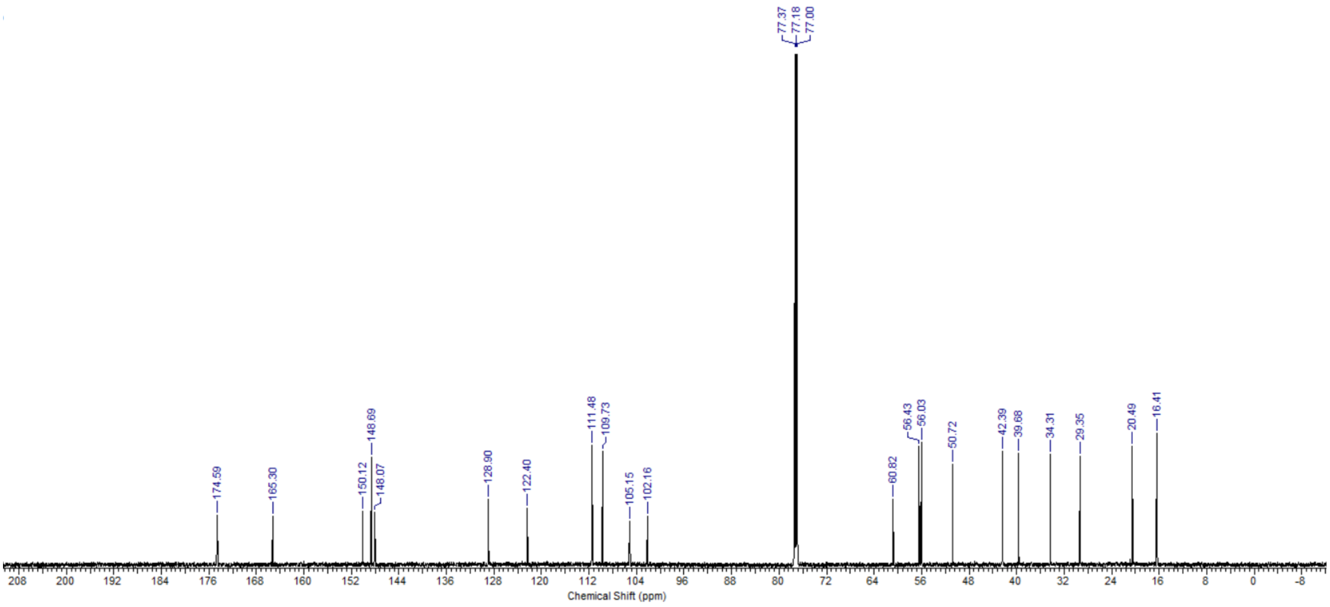

Methyl 3a-benzyl-10,11-dimethoxy-2-oxo-3,3a,7,8-tetrahydro-2*H*-furo[2’,3’:2,3]pyrrolo[2,1-*a*]isoquinoline-4-carboxylate 3c

^{1}H NMR (600 MHz, $CDCl_3$):

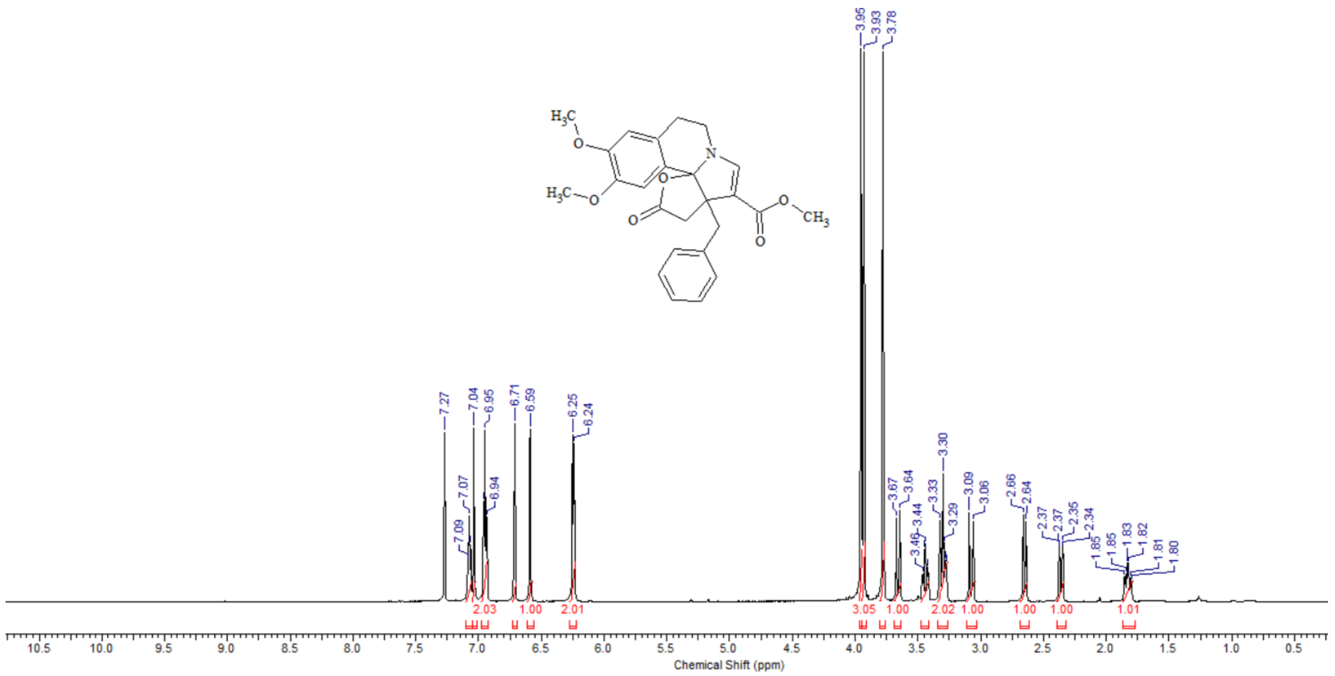

^{13}C NMR (150 MHz, $CDCl_3$):

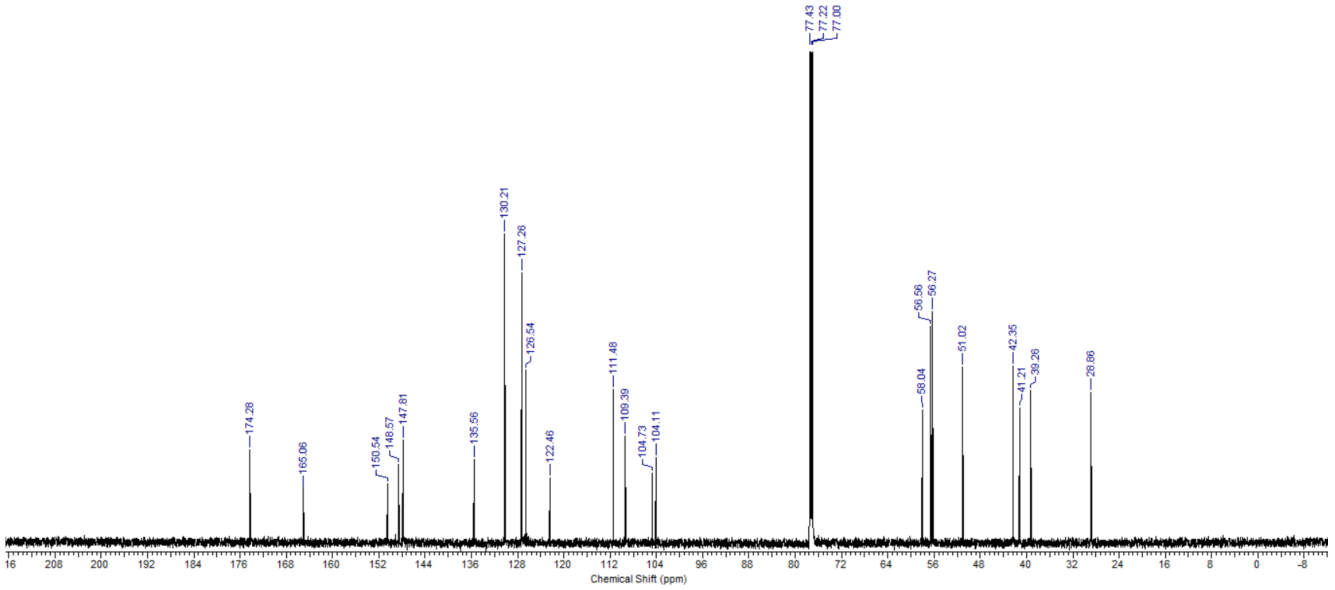

Methyl 10,11-dimethoxy-2-oxo-3a-phenyl-3,3a,7,8-tetrahydro-2*H*-furo[2’,3’:2,3]pyrrolo[2,1-*a*]isoquinoline-4-carboxylate 3d

^{1}H NMR (600 MHz, CDCl$_3$):

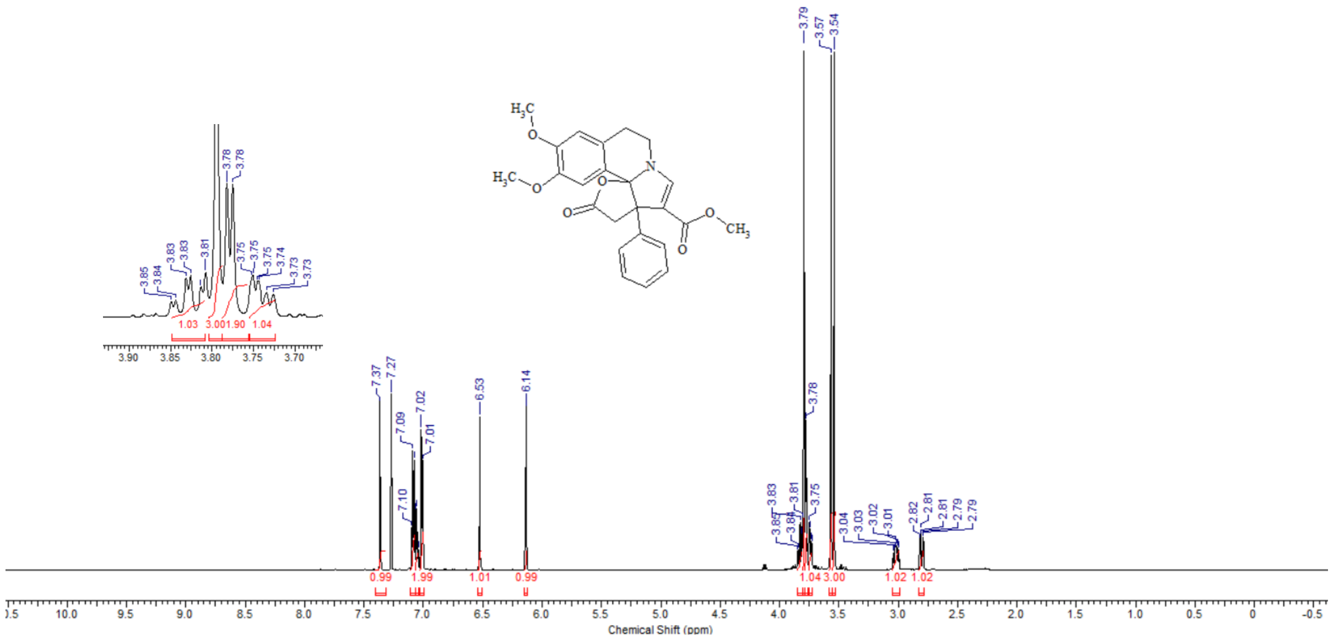

^{13}C NMR (150 MHz, CDCl$_3$):

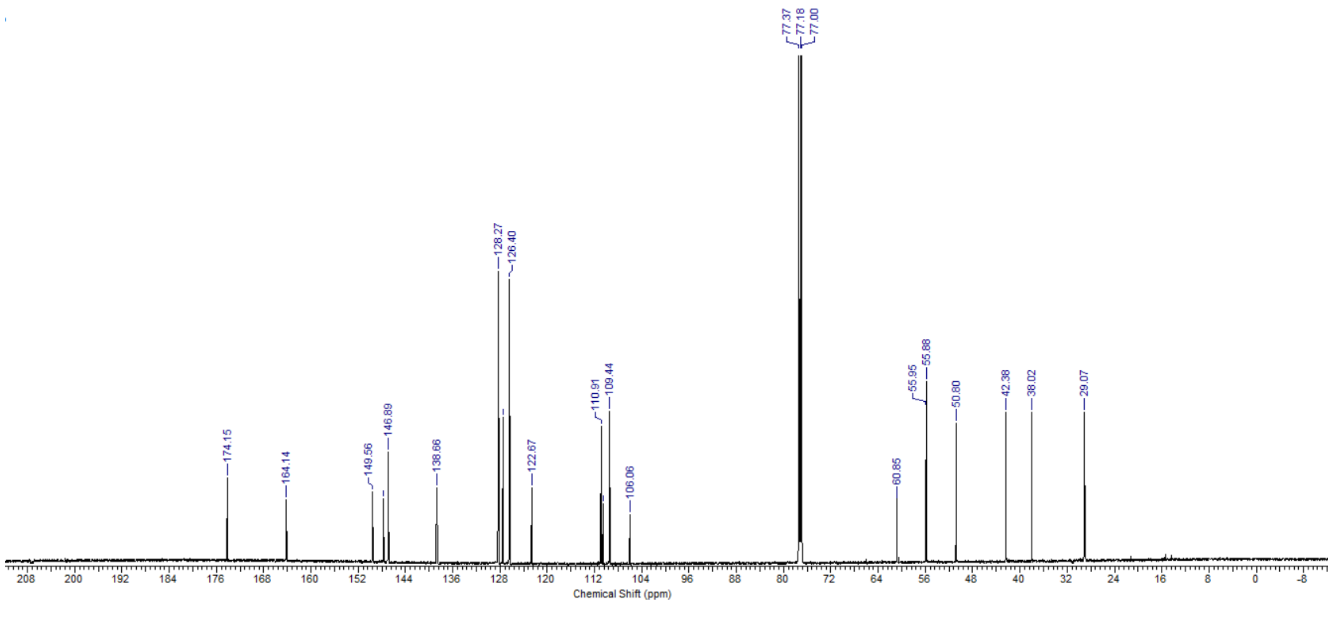

Methyl 10,11-dimethoxy-3a-(4-methoxyphenyl)-2-oxo-3,3a,7,8-tetrahydro-2*H*-furo[2’,3’:2,3]pyrrolo[2,1-*a*]isoquinoline-4-carboxylate 3e

^{1}H NMR (600 MHz, $CDCl_3$):

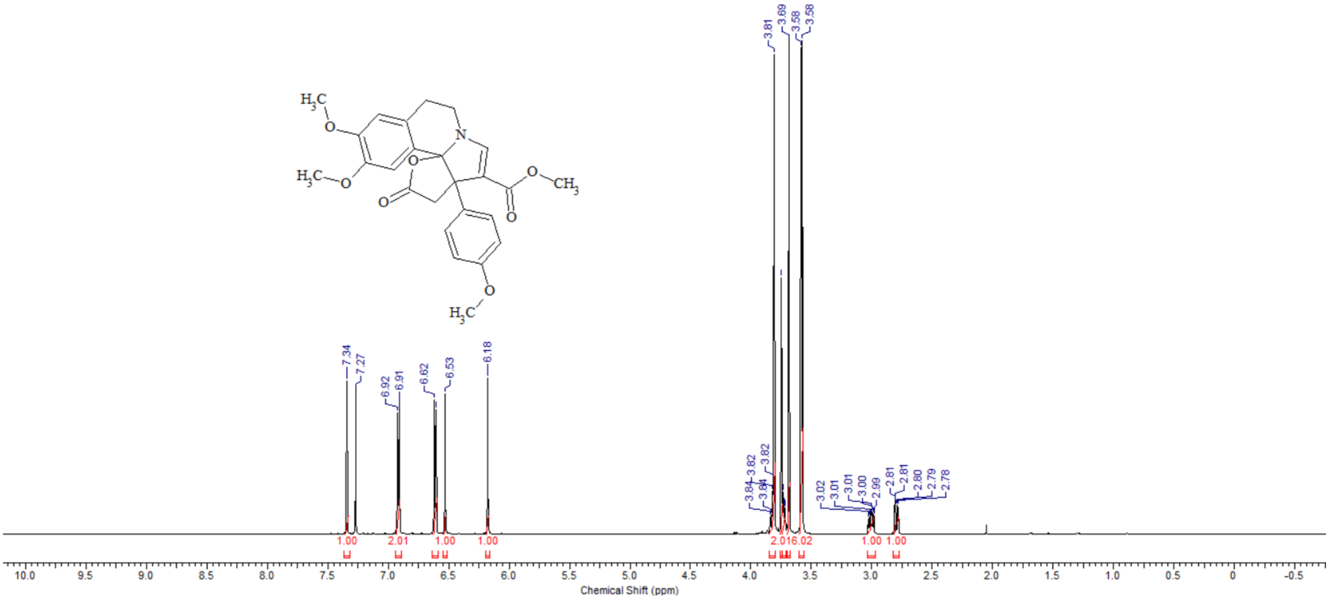

^{13}C NMR (150 MHz, $CDCl_3$):

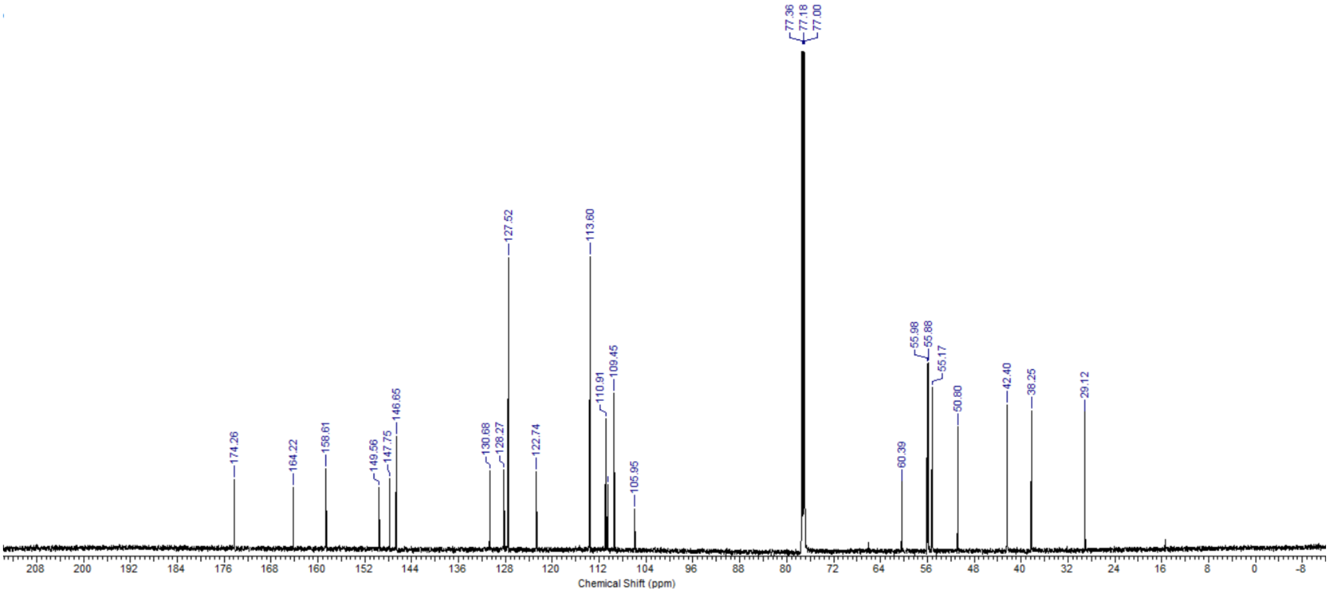

Methyl 3a-(4-fluorophenyl)-10,11-dimethoxy-2-oxo-3,3a,7,8-tetrahydro-2*H*-furo[2’,3’:2,3]pyrrolo[2,1-*a*]isoquinoline-4-carboxylate 3f

^{1}H NMR (600 MHz, $CDCl_3$):

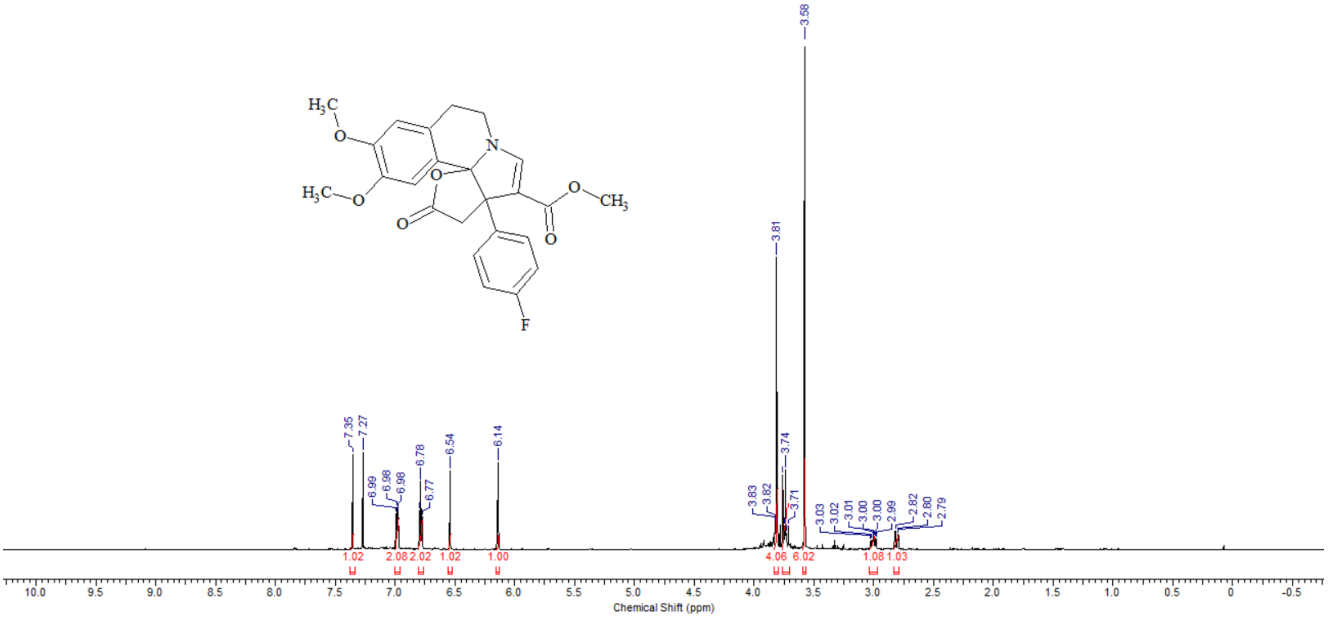

^{13}C NMR (150 MHz, $CDCl_3$):

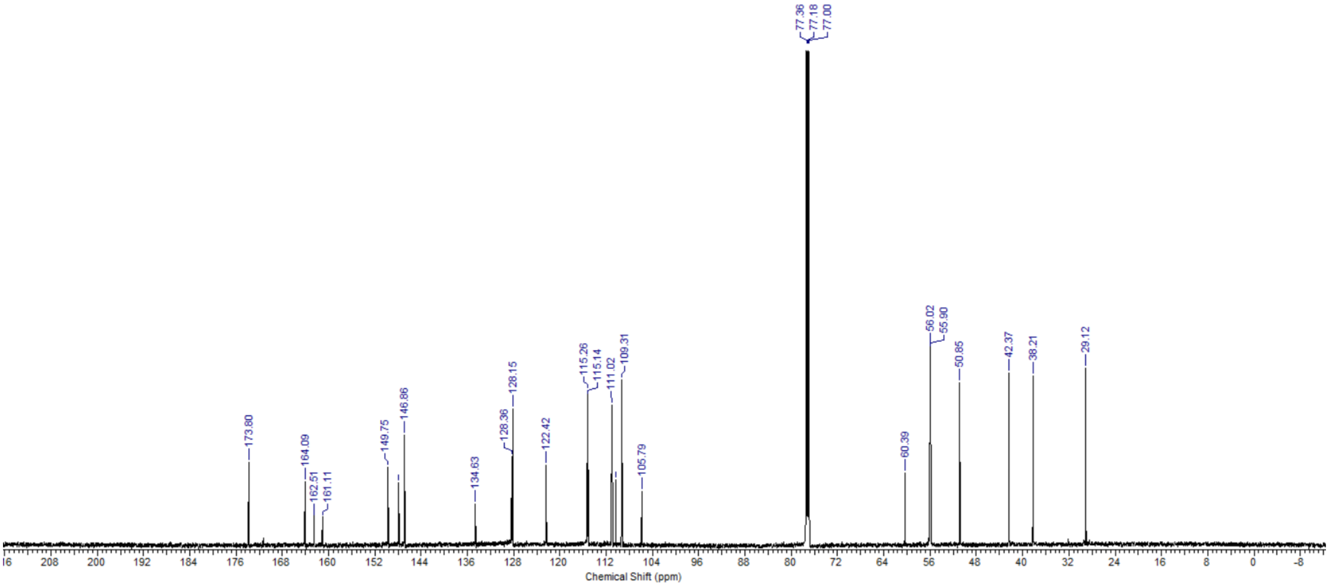

Methyl 10,11-dimethoxy-3a-(4-nitrophenyl)-2-oxo-3,3a,7,8-tetrahydro-2*H*-furo[2’,3’:2,3]pyrrolo[2,1-*a*]isoquinoline-4-carboxylate 3g

^{1}H NMR (600 MHz, $CDCl_3$):

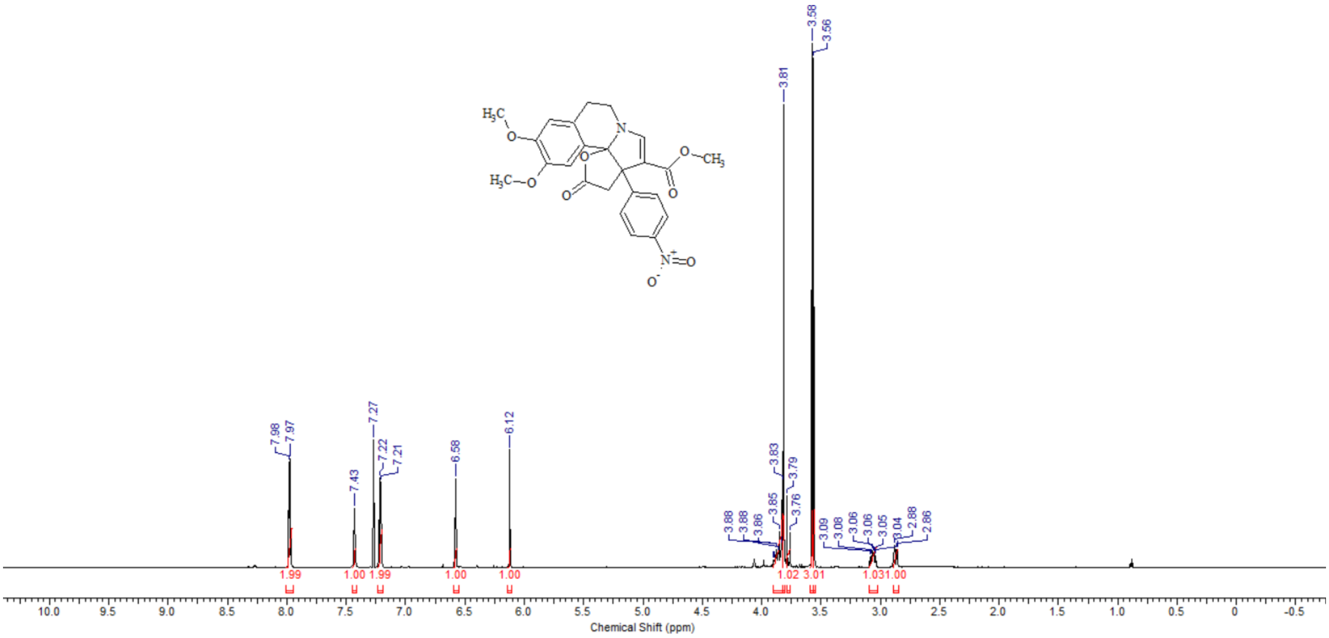

^{13}C NMR (150 MHz, $CDCl_3$):

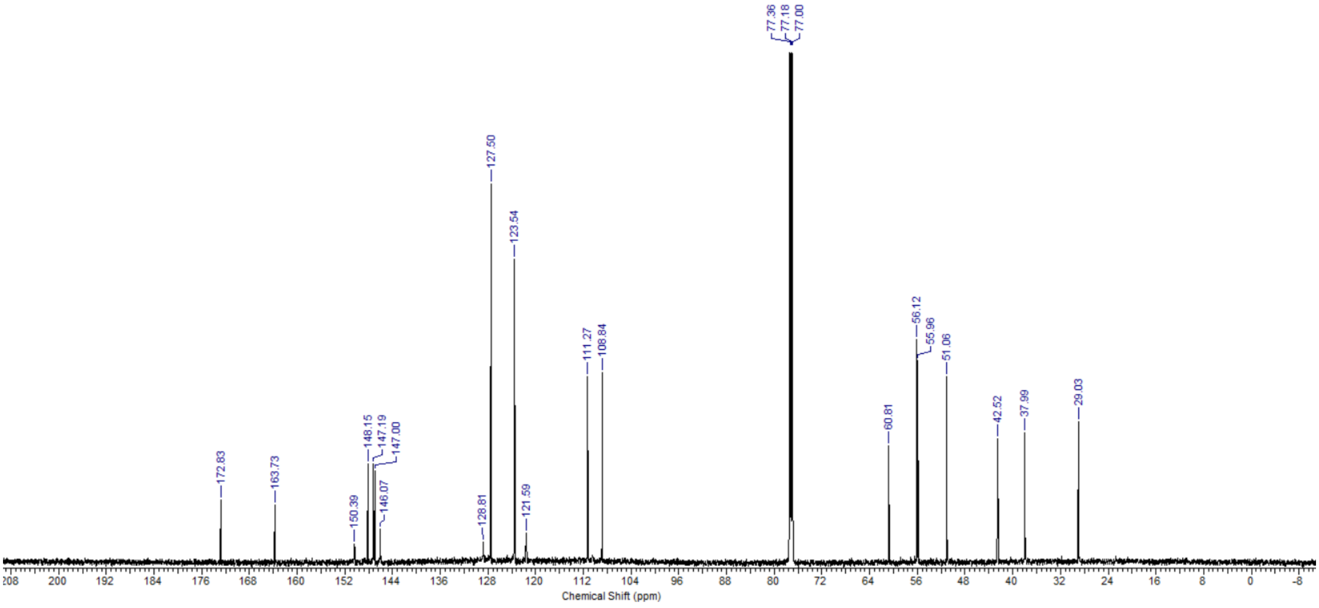

Dimethyl 11-hydroxy-8,9-dimethoxy-11-(4-methoxyphenyl)-6,11-dihydro-5*H*-pyrrolo[2,1-*b*][3]benzazepine-1,2-dicarboxylate 5a

^{1}H NMR (600 MHz, $CDCl_3$):

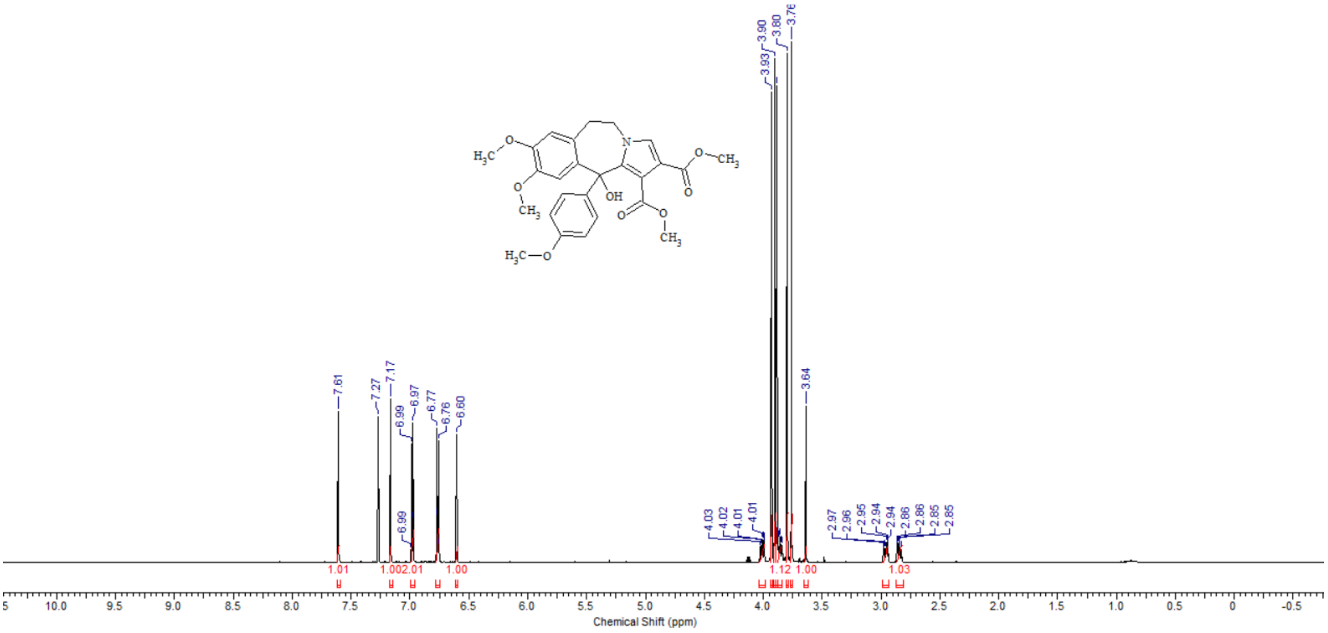

^{13}C NMR (150 MHz, $CDCl_3$):

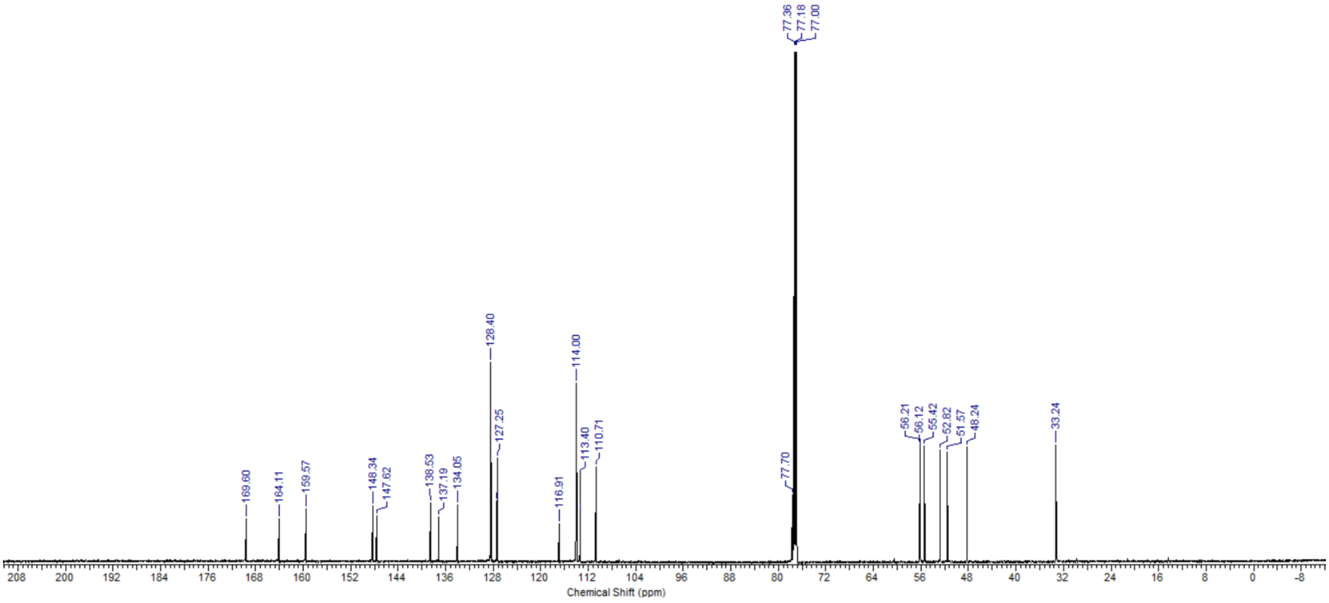

Dimethyl 11-(4-fluorophenyl)-11-hydroxy-8,9-dimethoxy-6,11-dihydro-5*H*-pyrrolo[2,1-*b*][3]benzazepine-1,2-dicarboxylate 5b

^{1}H NMR (600 MHz, $CDCl_3$):

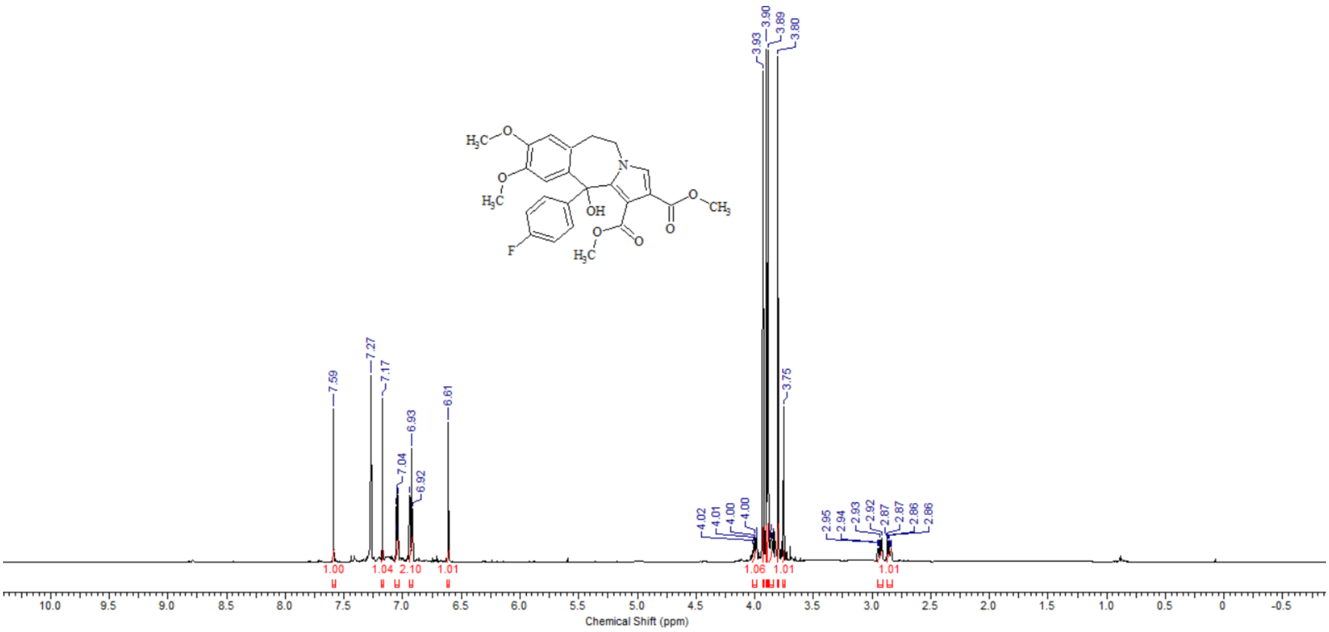

^{13}C NMR (150 MHz, $CDCl_3$):

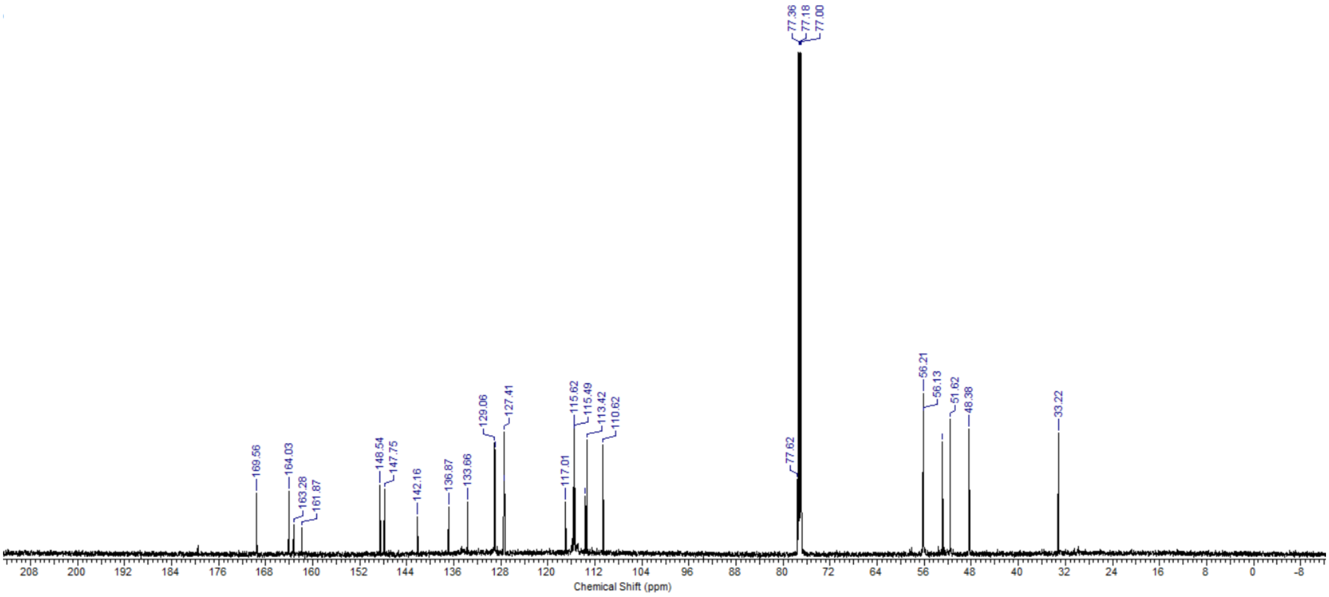

Methyl (1*E*)-8,9-dimethoxy-1-(2-methoxy-2-oxoethylidene)-10b-(4-methoxyphenyl)-1,5,6,10b-tetrahydropyrrolo[2,1-*a*]isoquinoline-2-carboxylate 6a

^{1}H NMR (600 MHz, $CDCl_3$):

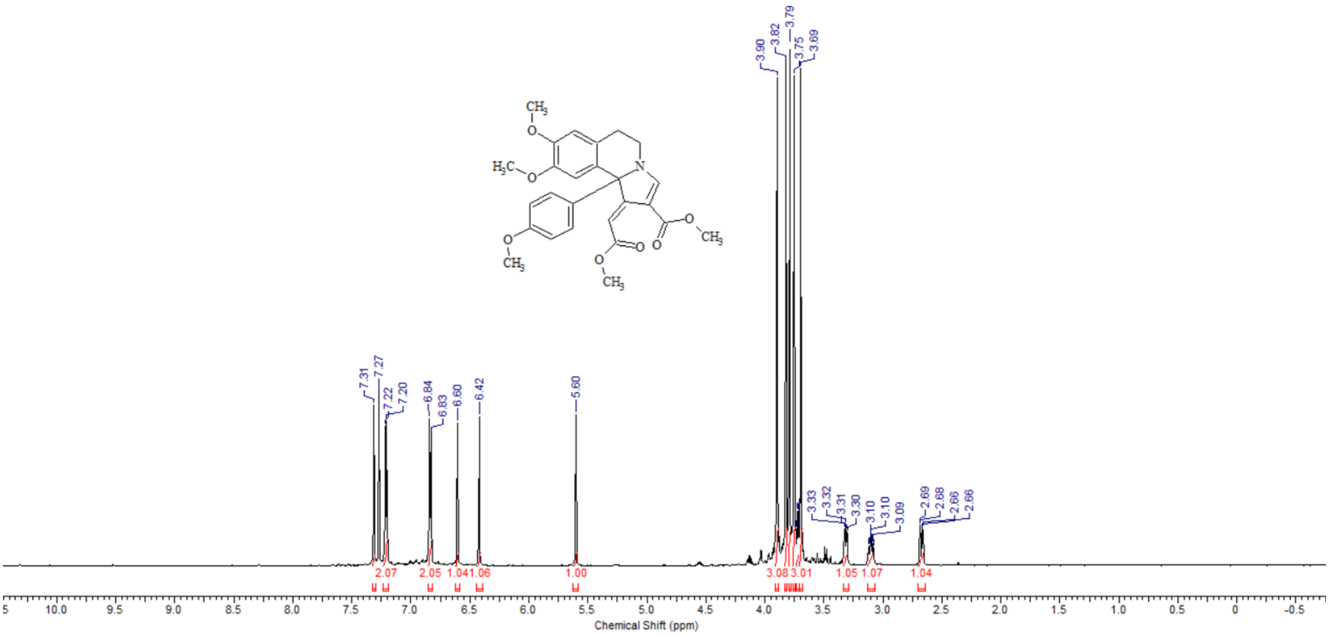

^{13}C NMR (150 MHz, $CDCl_3$):

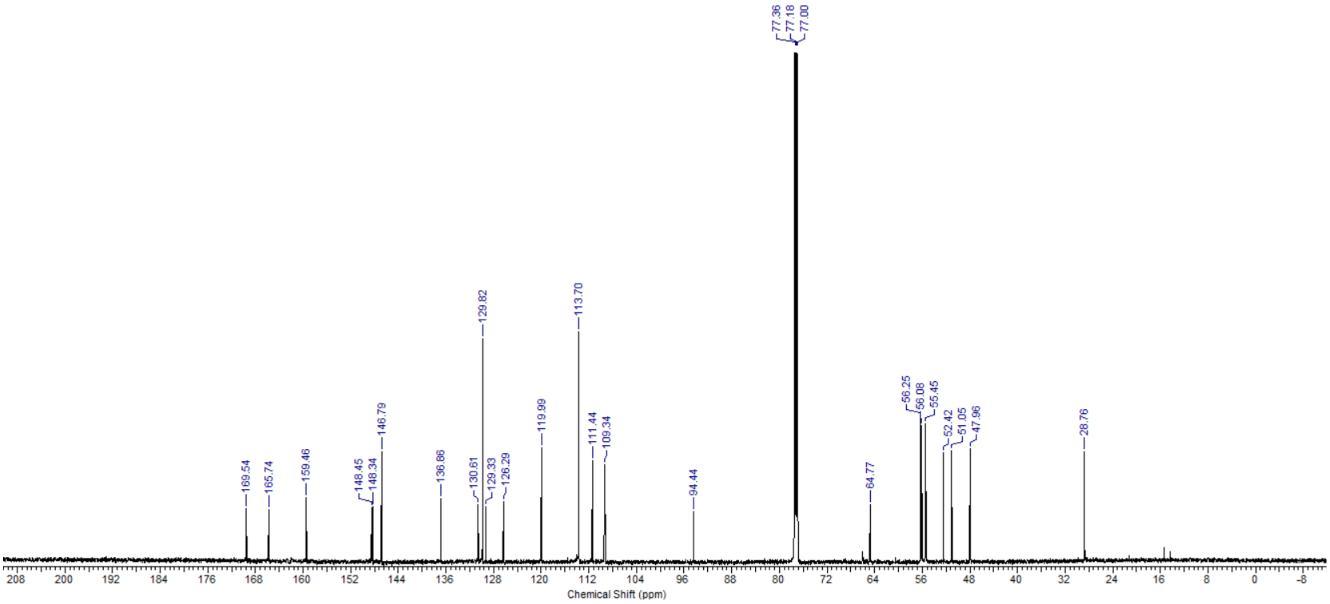

Methyl (1*E*)-8,9-dimethoxy-1-(2-methoxy-2-oxoethylidene)-10b-(4-nitrophenyl)-1,5,6,10b-tetrahydropyrrolo[2,1-*a*]isoquinoline-2-carboxylate 6b

^{1}H NMR (600 MHz, $CDCl_3$):

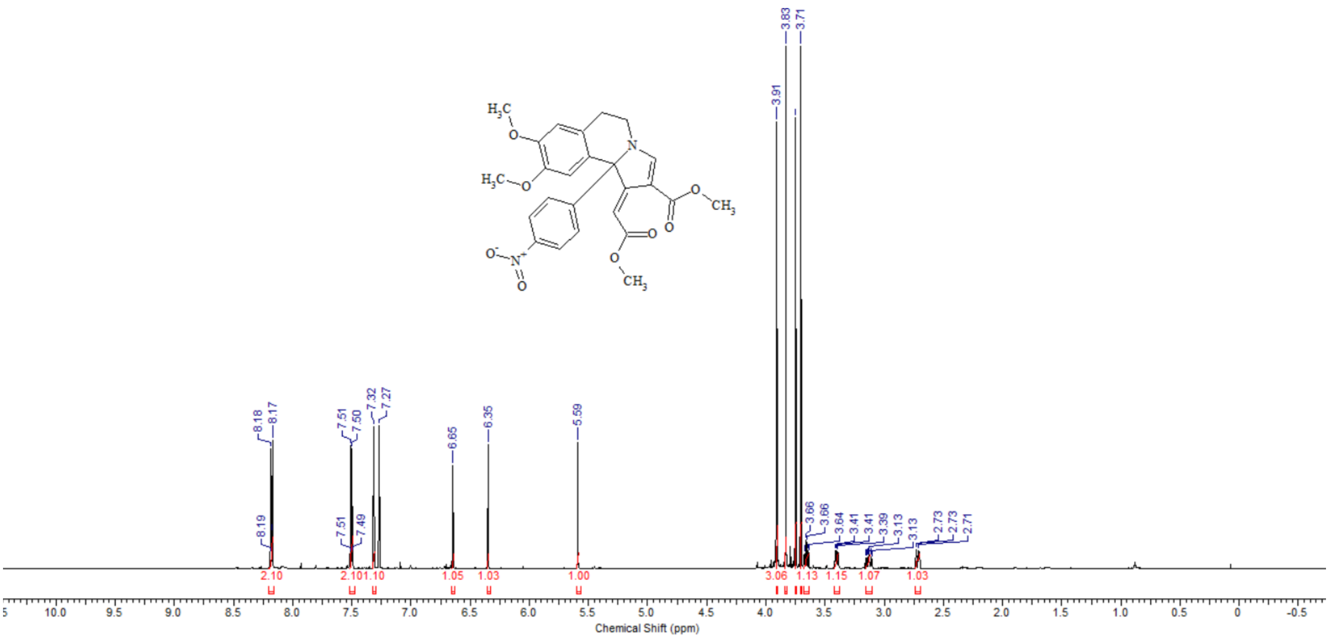

^{13}C NMR (150 MHz, $CDCl_3$):

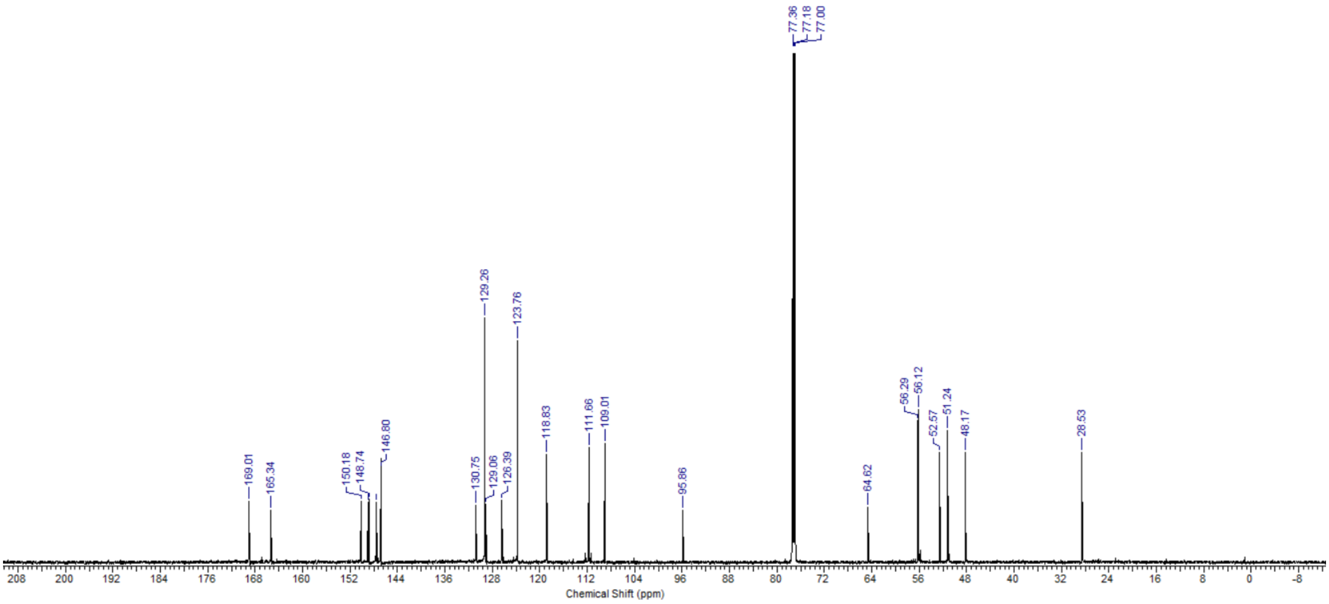

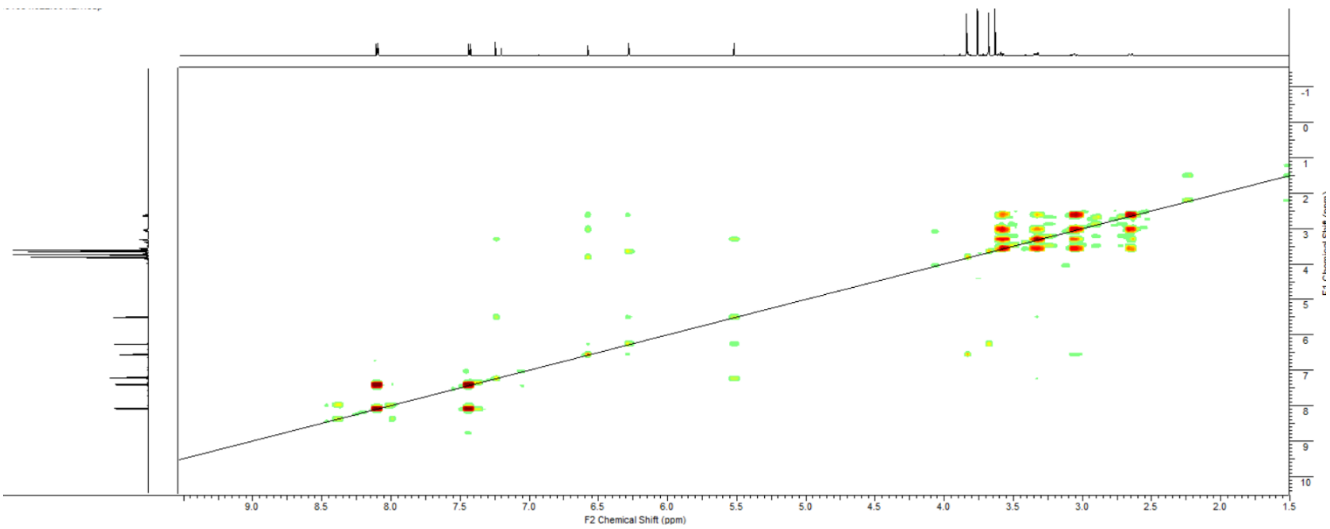

Figure S1. NOESY spectrum of **6b** in $CDCl_3$.

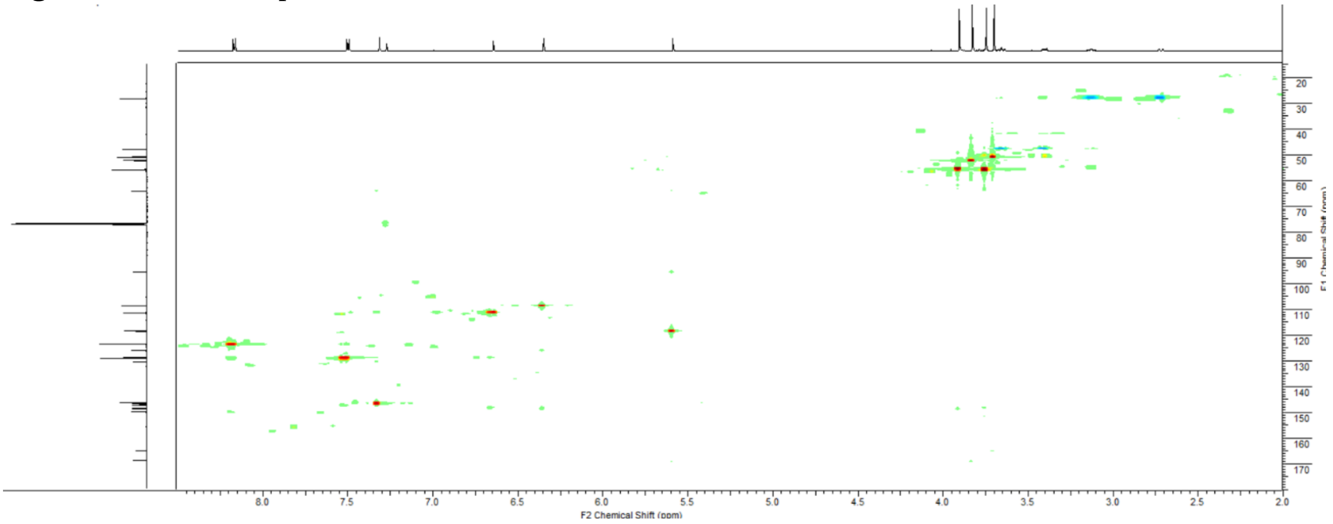

Figure S2. HMQC 1H-13C spectrum of **6b** in $CDCl_3$.

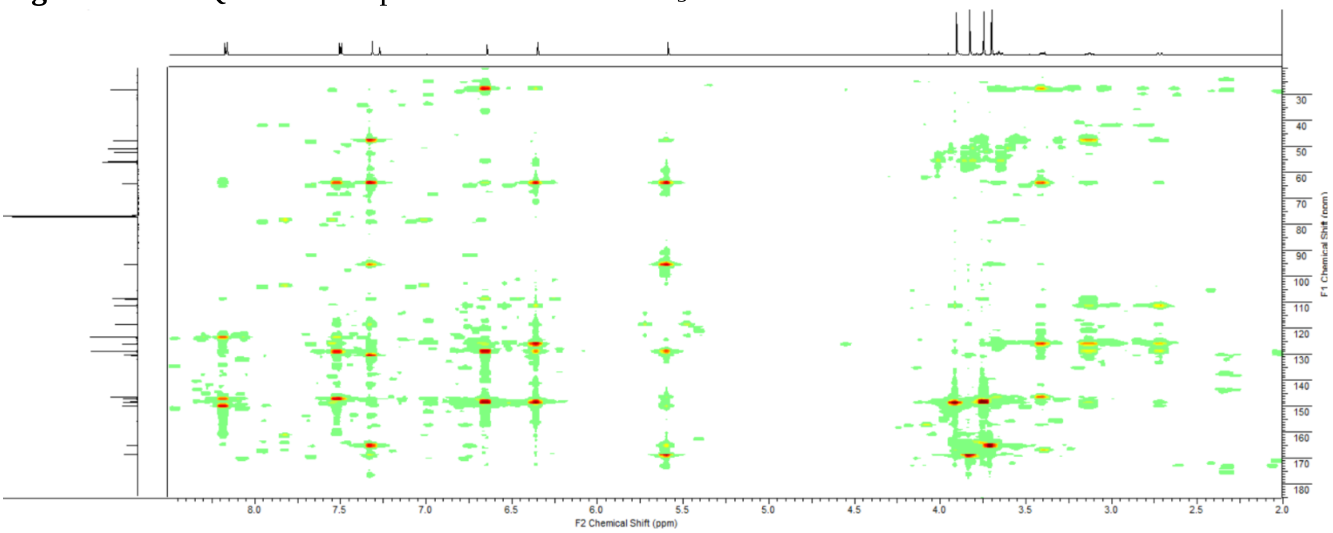

Figure S3. HMBC 1H-13C spectrum of **6b** in $CDCl_3$.
